# Utérus bicorne bicervical perméable: découverte fortuite lors d'une césarienne d'urgence chez une multipare lushoise

**DOI:** 10.11604/pamj.2013.15.75.2745

**Published:** 2013-06-25

**Authors:** Yves Idi Isango, Olivier Mukuku, Paul Makinko Ilunga, Christian Ngama Kakisingi, Joseph Nsambi, Christian Kabamba, Guy Mutangala, Patrick Kiopine Mubinda, Julien Kimbala

**Affiliations:** 1 Cliniques Universitaires de Lubumbashi, Faculté de Médecine, Université de Lubumbashi, RD Congo

**Keywords:** Utérus bicorne bicervical perméable, Utérus didelphe, Multipare, Lubumbashi, permeable bicervical uterus bicornis, uterus didelphys, multiparous, Lubumbashi

## Abstract

L'utérus bicorne bicervical perméable est une malformation utérine assez rare et son diagnostic est souvent posé fortuitement au cours d'un examen fait pour autre but. Il est asymptomatique et au cours d'une grossesse, cette malformation peut passer inaperçue si aucune surveillance prénatale n'a été faite. Nous rapportons un cas d'utérus bicorne bicervical perméable découvert fortuitement lors d'une césarienne indiquée urgemment pour une dystocie dynamique de type hypercinétique rebelle au traitement chez une multipare âgée de 30 ans. L'intérêt de ce cas est de montrer le pronostic obstétrical chez les femmes fertiles porteuses de cette malformation utérine.

## Introduction

La plupart de malformations congénitales sont décelables à la naissance, d'autres diagnostiquées en période anténatale et d'autres encore à la puberté ou l’âge adulte. Ces dernières passent souvent inaperçues pendant longtemps et ne sont découvertes que fortuitement. Parmi elles, nous retrouvons les malformations utérines en particulier les utérus bicornes bicervicaux qui peuvent être découverts lors d'un examen d'imagerie ou au cours d'une intervention chirurgicale abdominopelvienne pratiqués pour une autre raison, ou encore, comme dans notre cas, lors d'une césarienne effectuée pour une anomalie du travail ou de la présentation [1]. Nous rapportons ici un cas d'utérus bicorne bicervical perméable découvert fortuitement lors d'une césarienne indiquée urgemment pour une dystocie dynamique de type hypercinétique rebelle au traitement chez une multipare âgée de 30 ans. L'intérêt de ce cas est de montrer le pronostic obstétrical chez les femmes fertiles porteuses de cette malformation utérine.

## Patient et observation

Il s'agit d'une parturiente âgée 30 ans, P5G6A0D0, porteuse d'une grossesse de 39 semaines d'aménorrhée et 5 jours qui nous avait été transférée pour dystocie dynamique de type hypercinétique rebelle au traitement. Elle n'avait suivi aucune consultation prénatale; ses accouchements antérieurs étaient eutociques à terme et toutes les suites post-partales étaient simples. Son état général est bon et les signes vitaux sont dans les normes. L'examen obstétrical note une hauteur utérine de 35 centimètres, les bruits du c'ur f'tal perçus et comptés à 128 battements par minute, des contractions utérines de longue durée et d'intervalle rapproché, une dilatation cervicale de 7 centimètres, un effacement cervical complet, une absence de membranes, une présentation de siège décomplétée mode fesses non engagée. En plus, le toucher vaginal mettait en évidence un vagin non cloisonné et deux orifices cervicaux dont l'un était excentré vers la droite et l'autre central effacé et dilaté à 7 centimètres.

**Figure 1:**
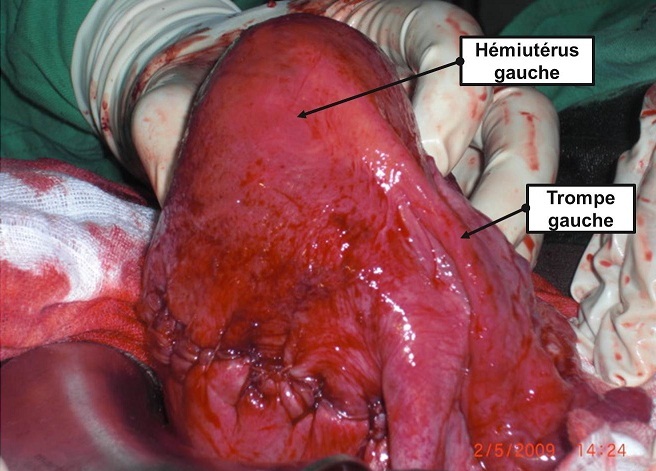
Hémi-utérus gauche après extraction du fœtus

**Figure 2:**
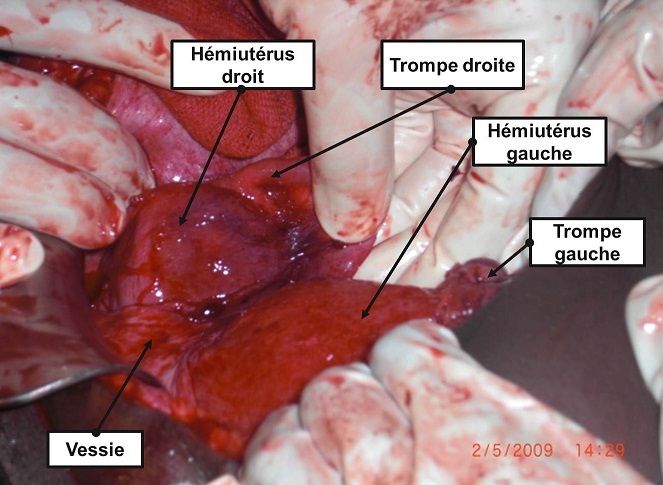
Constat per- opératoire après extration fœtale laissant voir la vessie, les deux hémi-utérus et leurs annexes

Une césarienne a été indiquée d'urgence et a permis l'extraction d'un nouveau-né masculin de 3200 grammes avec un score d'Apgar excellent. Le constat per- opératoire, après extraction f'tale, laisse objectiver deux hémi-utérus bien distincts totalement séparés comportant deux corps entre lesquels s'insinue la paroi vésicale postérieure (signe du V vésical) comportant chacun des annexes (ovaire, trompe, ligaments rond et large) d'un seul côté, ainsi que deux isthmes et deux cols. Le premier, à gauche, est gravide avec un segment inférieur bien formé et un hématome linéaire tranversale corporéal à la hauteur de l'insertion du ligament rond gauche au niveau du tiers droit de la face antérieure de l'utérus (rupture utérine partielle); le second, à droite, est non gravide augmenté volume et globuleux comparable à une grossesse de 8 à 10 semaines d'aménorrhée avec des annexes du côté droit (Figure 1, Figure 2). En per- opératoire, deux hystéromètres ont été introduits, l'un à travers un col dont l'ouverture ne permettait que le passage de la pulpe d'un doigt laissant passer facilement celui-ci et l'autre à travers un col dilaté à 7 centimètres. Le premier était introduit et prenait la direction droite à une profondeur de 13 centimètres et le second était visible au travers de l'incision de l'hystérotomie. Les suites post-opératoires étaient simples et la patiente est sortie au 7ème jour dans un bon état clinique.

Une exploration radiologique et échographique à la recherche des malformations rénales associées était programmée mais la patiente a été perdue de vue et ne s'est jamais présentée.

## Discussion

La prévalence des anomalies utérines congénitales dans la population est estimée entre 1 et 4% selon les études [1–3]. Il est difficile de déterminer la prévalence exacte puisque beaucoup de ces malformations sont asymptomatiques et semblent être diagnostiquées plus fréquemment chez les patientes suivies pour infertilité ou pour fausses couches à répétition. Les utérus bicornes bicervicaux perméables ou « utérus didelphes » des Anglosaxons, sont assez rares et leur fréquence varie selon les auteurs de 11 à 24,2% de l'ensemble des malformations utérines majeures [1, 4–6].

Sur le plan embryologique, ils sont dus à un défaut de fusion totale des canaux de Müller entre la 10^ème^ et la 13^ème^ semaine de grossesse aboutissant à la formation de deux cavités utérines non communicantes [2]. Un septum vaginal est absent dans 25% des cas comme c'est le cas dans notre observation. Selon la classification de l'American Fertility society, les utérus bicornes bicervicaux correspondent à la classe III. Ils sont accompagnés de malformations urinaires unilatérales à type d'agénésie dans 10 à 50% des cas [3, 7].

S'agissant de la clinique, dans beaucoup de cas, les utérus bicornes bicervicaux restent asymptomatiques surtout s'ils sont perméables; le diagnostic n'est posé que fortuitement lors d'un examen pratiqué dans un autre but. Ainsi le diagnostic d'un utérus bicorne bicervical peut être posé lors d'un premier contrôle de grossesse ou de découvrir lors d'un accouchement par voie basse la présence d'un septum vaginal ou de deux cols méconnus jusqu'alors [3]. Etant donné sa rareté, l’âge et la multiparité de la patiente, il était surprenant que cette anomalie n'ait jamais était détectée au cours des surveillances prénatales des grossesses antérieures.

Du point de vue obstétrical, dans l'ensemble, les malformations utérines sont pourvoyeuses d'un grand nombre de présentations dystociques, et parmi ces dernières, une présentation du siège est retrouvée dans 23 à 61% des cas de malformation utérine [1]; quant au mode d'accouchement, la fréquence des césariennes est significativement plus élevée en cas de malformation utérine, avec des taux compris entre 27,5 et 83% selon les auteurs [1, 5, 8], ceci s'explique par le fait que les malformations utérines sont souvent associées à des présentations dystociques, mais elles sont également associées à une plus grande fréquence d'anomalies du travail dans environ 50% des cas, à type de dystocies cervicales et dynamiques [1]. En ce qui concerne notre patiente, aucune complication obstétricale n'avait été observée auparavant lors de ses cinq précédents accouchements. Ce n'est qu'au cours de la sixième grossesse qu'elle a pu présenter une hypercinésie rebelle au traitement, sanctionnée par une césarienne. Certains types de malformations utérines ont des conséquences sur la vie reproductrice et exigent la chirurgie pour rétablir la continuité. Mais en cas d'utérus bicorne bicervical perméable, la chirurgie réunificatrice de deux hémi-utérus est réservée aux patientes dont le pronostic obstétrical est très mauvais.

## Conclusion

Les malformations utérines sont fréquentes dans la population générale mais les conséquences sur la reproduction varient suivant le type de malformation. Vu les antécédents et l'identité obstétricaux de notre patiente, nous pouvons dire que le pronostic obstétrical chez les femmes porteuses d'utérus bicorne bicervical perméable semble souvent être très bon.
